# Performance of the Roche Elecsys® IGRA SARS-CoV-2 test for the detection and quantification of virus-reactive T cells in COVID-19-vaccinated immunosuppressed patients and healthy subjects

**DOI:** 10.1007/s10096-024-04852-5

**Published:** 2024-05-23

**Authors:** Diego Carretero, Estela Giménez, Eliseo Albert, Ester Colomer, Marco Montomoli, Rafael Hernani, José Luis Piñana, José Luis Górriz, Carlos Solano, David Navarro

**Affiliations:** 1 Microbiology Service, Clinic University Hospital, INCLIVA Health Research Institute, Valencia, Spain; 2 Nephrology Service, Hospital Clínico Universitario de Valencia, INCLIVA Health Research Institute, Valencia, Spain; 3 Hematology Service, Hospital Clínico Universitario, INCLIVA Health Research Institute, Valencia, Spain; 4 Department of Medicine, School of Medicine, University of Valencia, Valencia, Spain; 5 Department of Microbiology, School of Medicine, University of Valencia, Valencia, Spain; 6 Centro de Investigación Biomédica en Red de Enfermedades Infecciosas (CIBERINFEC), Instituto de Salud Carlos III, Madrid, Spain; 7 Microbiology Service, Hospital Clínico Universitario, Instituto de Investigación INCLIVA, Av. Blasco Ibáñez 17, 46010 Valencia, Spain

**Keywords:** SARS-CoV-2, Interferon-gamma release assay (IGRA), T cells, Flow cytometry for intracellular staining, COVID-19

## Abstract

**Purpose:**

Comparing the performance of commercially available SARS-CoV-2 T-cell immunoassay responses may provide useful information for future observational or intervention studies as well as to their potential customers.

**Method:**

Whole blood was collected from a total of 183 subjects fully vaccinated against COVID-19: 55 healthy controls (Group 1), 50 hematological patients (Group 2), 50 chronic kidney disease patients (Group 3), and 28 elderly nursing home residents (Group 4). Samples were tested with the Roche Elecsys® IGRA (Interferon-gamma release assay) SARS-CoV-2 test (Roche Diagnostics, Rotkreuz, Switzerland), the Euroimmun SARS-CoV-2 test (Euroimmun, Lubeck, Germany), the SARS-CoV-2 T Cell Analysis Kit (Miltenyi Biotec, Bergisch Gladbach, Germany), and a flow-cytometry for intracellular cytokine (IFN-γ) staining-based immunoassay (FC-ICS).

**Results:**

Overall, the Roche Elecsys® assay returned the highest number of positive results (151/179; 84.3%), followed by the Euroimmun test (127/183; 69%), and the FC-ICS (135/179; 75%). The Kappa coefficient of agreement was best between IGRAs (0.64). Most discordant results across assays involved patients from Group 2. Overall, IFN-γ concentrations measured by both IGRAs correlated strongly (rho = 0.78; 95% CI 0.71–0.84; *P* < 0.001) irrespective of the study group. The frequencies of SARS-CoV-2-reactive IFN-γ T cells and IFN-γ concentrations measured by the IGRAs correlated moderately for CD4^+^ T cells, however, weakly for CD8^+^ T cells. SARS-CoV-2-experienced participants displayed stronger responses than SARS-CoV-2-naïve when IGRAs, rather than FC-ICS, were used.

**Conclusion:**

The SARS-CoV-2 immunoassays evaluated in the present study did not return interchangeable qualitative or quantitative results either in seemingly healthy individuals or in immunosuppressed patients.

## Introduction

Both innate and adaptive (B and T-cell mediated) immune responses are critical in the control of SARS-CoV-2 replication [1–3]. Following natural infection, delayed and dysregulated interferon-mediated responses are associated with sustained inflammation and poor clinical outcomes [4–6], whereas timely activation of functional SARS-CoV-2-reactive T cells seemingly prevents the development of severe COVID-19 [2, 3, 7]. Memory T cells, recognizing a broad spectrum of epitopes mapping within structural (membrane [M], nucleocapsid [N], and non-structural [nsp]) proteins in addition to the Spike (S) protein, are targeted following natural infection. However, immunogenic epitopes within the S1 and S2 components of the SARS-CoV-2 Spike protein develop after vaccination with mRNA-based licensed platforms [1–3, 8–10]. Vaccine-elicited SARS-CoV-2-S-reactive T cells are paramount in providing protection against severe COVID-19 [11]. Currently, circulating SARS-CoV-2 Omicron subvariants substantially evade recognition by neutralizing antibodies elicited by Wuhan-Hu-1-based vaccines or prior infection by preceding subvariants. In contrast, induced T-cell responses appear to cross-recognize these subvariants with almost similar efficiency, thus avoiding significant viral escape [2, 12–18]. Evaluation of SARS-CoV-2 T-cell immunity in whole blood specimens may help to identify individuals at the highest risk of severe COVID-19 and assess the need for booster vaccine doses, both of particular clinical relevance in immunosuppressed individuals, who are frequently lymphopenic or unable to develop functional T-cell responses [3, 17, 18]. However, no T-cell thresholds for protection against SARS-CoV-2 infection or severe COVID-19 have been defined. Flow cytometry for intracellular cytokine staining (FC-ICS) or activation-induced markers allows sensitive detection, precise quantification, and functional characterization of SARS-CoV-2-reactive T cells; nevertheless, these procedures lack robust standardization and require high technical expertise [18]. Several commercial platforms have been developed to evaluate SARS-CoV-2 T-cell immunity [18]; these are based on the detection and quantification of IFN-γ produced by peripheral effector T cells after antigenic stimulation (Interferon-gamma release assays—IGRA) by enzyme-linked immunosorbent or immunospot (ELISpot) assays. A limited number of studies have made head-to-head comparisons of the performance of different commercially available IGRAs and flow cytometry-based assays [19–22]. This seems relevant as results from the aforementioned studies strongly suggest that the qualitative and quantitative results provided by these platforms are not interchangeable. Moreover, these studies may provide useful information to potential customers of SARS-CoV-2 T-cell immunoassays. Here, we compared the qualitative performance of the Roche Elecsys® IGRA SARS-CoV-2 test (Roche Diagnostics, Rotkreuz, Switzerland) with that of the Euroimmun SARS-CoV-2 test (Euroimmun, Lubeck, Germany) [19] and the SARS-CoV-2 T Cell Analysis Kit-Whole blood- (Miltenyi Biotec, Bergisch Gladbach, Germany), and a flow cytometry for intracellular cytokine (IFN-γ) staining-based immunoassay, which is currently used in our laboratory for assessing SARS-CoV-2-S T-cell responses in vaccinated individuals. Moreover, we investigated to what extent the quantitative values returned by these immunoassays correlated. To this end, in addition to healthy individuals, we recruited elderly subjects and immunosuppressed patients, who frequently display suboptimal T-cell responses to licensed vaccines [22]. To our knowledge, no previous studies have compared the performance of the Roche Elecsys® IGRA SARS-CoV-2 test with that of other commercially available IGRA or flow cytometry-based immunoassays.

## Material and methods

### Subjects and samples

A convenient series of 183 subjects were enrolled in the current observational, prospective, cross-sectional, unicenter study. These were categorized into four population groups: Group 1 included 55 healthy control individuals (mean age, 38 years; range, 19–63); Group 2, comprised 50 hematological patients (mean age, 58 years; range, 26–83), of whom 24 received an allogeneic hematopoietic stem-cell transplant, 16 had a non-transplanted hematological malignancy, 6 underwent chimeric antigen receptor T-cell therapy (CAR-T), and 4 underwent autologous hematopoietic stem-cell transplantation; Group 3 included 50 patients (mean age, 64 years; range 26–83) with chronic kidney disease (n = 26) or kidney transplantation (n = 24); and Group 4 comprised 28 elderly nursing home residents (mean age, 87 years; range, 68–99). Overall, 46% of participants were male and 54% female. All participants had been fully vaccinated (two doses) with licensed mRNA SARS-CoV-2 vaccines. Some participants from Group 1 and all participants from Groups 2,3, and 4 had received two booster vaccine (mRNA-based) doses. Whole blood samples were collected from each participant at a single time point in two lithium heparin tubes for IGRA and in one sodium heparin tube for FC-ICS. IGRAs and FC-ICS were performed in parallel within six hours of blood extraction. The median time from the last vaccine dose to immunological testing was 200, 270, 300, and 210 days for participants in Groups 1,2,3, and 4, respectively. The study was conducted between May and November 2023 and was approved (2022/351) by the Institutional (INCLIVA) Ethics Committee. All subjects agreed to voluntarily participate in the study and gave written consent.

### Serological assay

Detection of SARS-CoV-2-nucleocapsid (N)-reactive total antibodies in plasma was carried out by the Roche Elecsys® Anti-SARS-CoV-2 N assay (Roche Diagnostics, Pleasanton, CA, USA). Plasma for serological analyses was available from 173 participants. Since anti-SARS-CoV-2 N-reactive antibodies cannot be elicited following S-based mRNA vaccine platforms, participants with detectable anti-N antibodies were categorized as SARS-CoV-2 experienced.

### Elecsys® IGRA SARS-CoV-2 assay (Roche Diagnostics, Rotkreuz, Switzerland)

Whole blood samples in lithium heparin tubes (Franklin Lakes, New Jersey, USA) were transferred to Negative Control (NC), Antigen (Ag), and Positive Control (PC) tubes (1.2 ml per tube), inverted 20 times, and incubated at 37ºC for 20 h. After incubation, plasma was separated by centrifugation and used for IFN-γ quantification by using the Electrochemiluminescent (ECLIA) Elecsys® IGRA SARS-CoV-2 test in the Cobas® e 411 analyzer (Roche Diagnostics, Rotkreuz, Switzerland), according to the manufacturer’s recommendations. The Ag tube contained a total of 189 structural and non-structural SARS-CoV-2 immunogenic peptides targeted by CD4^+^ and CD8^+^ T cells. The IFN-γ concentration in each sample was calculated as that measured in the Ag tube minus that quantified in the NC tube. The cut-off for positivity was established by the manufacturer at 0.013 IU/ml. The test was considered invalid if the IFN-γ concentration in the PC tube was < 1.0 IU/ml; the result was deemed indeterminate if the IFN-γ concentration in the NC tube was > 0.3 IU/ml.

### Euroimmun SARS-CoV-2 IGRA test (Euroimmun, Lubeck, Germany)

A volume of 0.5 ml of whole blood collected in lithium tubes (Franklin Lakes, New Jersey, USA) was transferred into each of the following tubes: CoV-2 IGRA BLANK (background), CoV-2 IGRA (containing antigens mapping within the S1 domain of the SARS-CoV-2 Spike protein), and CoV-2 IGRA STIM (mitogen). The tubes were inverted several times and incubated at 37ºC for 20 h. After incubation, plasma was obtained by centrifugation and stored at -20ºC until analyzed. The Quan-T-Cell ELISA test (Euroimmun, Lubeck, Germany) was used for IFN-γ concentration measurement according to the manufacturer’s recommendations. The final IFN-γ concentration for a given sample was calculated using a standard curve built from each experiment after subtracting the IFN-γ concentration obtained in the BLANK tube. Results were interpreted according to the manufacturer’s recommendations: < 100 mIU/ml negative; 100–200 mIU/ml, borderline, and > 200 mIU/ml positive. For some analyses, mIU/ml was expressed as IU/ml.

### SARS-CoV-2 flow cytometry immunoassay for intracellular IFN-γ staining

The frequency of SARS-CoV-2-reactive IFN-γ-producing CD4^+^ or CD8^+^ T cells was determined with the SARS-CoV-2 T Cell Analysis Kit-Whole blood- (Miltenyi Biotec, Bergisch Gladbach, Germany). All reagents detailed below, except when indicated, were included in the kit. Briefly, whole blood (1.5 ml) collected in sodium heparin tubes (Franklin Lakes, New Jersey, USA) was transferred into three tubes, Antigen (Ag), negative control (NC), and positive control (PC), and stimulated (Ag) with 50 µL of Peptivator SARS-CoV-2 S protein, a pool of lyophilized peptides that covers the entire protein sequence of the Wuhan-Hu-1 Spike protein. The NC tube was mock-stimulated with dimethyl sulfoxide. The PC tube was stimulated with the superantigen Cytostim™ human. A volume of 20 µL of Brefeldin A was then added to each tube and samples were incubated at 37ºC 5% CO_2_ for 12 h. After incubation, Red Blood Cell Lysis Solution was added and, after a washing step with autoMACS Running Buffer (PEB buffer; PBS, pH 7.4, 0.5% bovine serum albumin, and 2 mM EDTA), samples were fixed with Fixation Mix. After two additional washing steps with autoMACS Running Buffer, 1 mL of StemMACS Cryo-Brew was added, and samples were kept at -80ºC until analysis by flow cytometry. On the day of analysis, samples were thawed, washed twice with PEB buffer, and 1 mL of Inside Perm was added. After centrifugation, 100 µL of antibody staining cocktail (CD3-APC, CD4-FITC, CD8-BV510, IFN-γ PE) was added to each sample and incubated for 10 min in the dark. The monoclonal antibody clones (Miltenyi Biotec) used were the following: CD3 + (REA613) CD4 + (REA623), CD8 + (REA734), and IFN-µ (REA600). After incubation, an additional 1 mL of Inside Perm was added and samples were centrifuged. The resultant cell pellet was resuspended in 250 µL of PEB buffer and samples were acquired in an LSRFortessa™ flow cytometer (BD Biosciences Immunocytometry Systems, San Jose, CA) using FlowJo v-10 software (BD Biosciences). CD3^+^/CD8^+^ and CD3^+^/CD4^+^ events were gated and then analyzed for IFN-γ production. All data were corrected for background IFN-γ production (NC tube) and expressed as the number of SARS-CoV-2-reactive IFN-γ-producing CD4^+^ or CD8^+^ T cells relative to the absolute number of CD4^+^ and CD8^+^ T cells, respectively, × 100 (%). Any frequency value of SARS-CoV-2-reactive IFN-γ-producing CD4^+^ or CD8^+^ T cells after background subtraction was considered a positive (detectable) result and used for analytical purposes.

### Statistical analyses

Positive percent agreement (PPA) and negative percent agreement (NPA) values are reported throughout the study. Cohen’s kappa test was used for assessing the level of agreement across assays and was interpreted as follows: kappa = 0.10–0.20 slight agreement; kappa = 0.21–0.40 fair agreement; kappa = 0.41–0.60: moderate agreement; kappa = 0.61–0.80: substantial agreement, and Kappa = 0.81–0.99: perfect agreement. McNemar’s test was applied to analyses of paired categorical data. Comparison of continuous variables across two or more groups was carried out with the non-parametric Mann–Whitney U test for unpaired variables, and the Wilcoxon test for paired variables, as appropriate. The association between two continuous variables was analyzed using the non-parametric Spearman correlation test. Rho values between 0.8–1 were regarded as very strong, between 0.6–0.79, strong, between 0.40–0.59, moderate, between 0.2–0.39, weak, and below 0.20, very weak. Receiver operating characteristic (ROC) analysis was used to select the best cut-off for Euroimmun-IGRA positivity using the Elecsys® assay as a reference. *P* values < 0.05 were deemed statistically significant. Statistical analyses and graphs were performed using GraphPad Prism version 10.0.0 for Windows.

## Results

### SARS-CoV-2 infection status of participants

Plasma for serological analyses was available from 173 participants (95%). A sufficient volume of plasma for analysis was not available from the remaining 10 participants. Based upon the detection of SARS-CoV-2 anti-N, a total of 146 participants (84%) were categorized as SARS-CoV-2-experienced and 27 (16%) as SARS-CoV-2-naïve. SARS-CoV-2-naïve individuals were differently represented across study groups (Group 1, n = 2; Group 2, n = 5; Group 3, n = 17; and Group 4, n = 3).

### Qualitative performance comparison between the Roche Elecsys® IGRA SARS-CoV-2 assay and the Euroimmun SARS-CoV-2 IGRA test

Data on the performance of the Roche Elecsys® assay were available from 181 of the 183 subjects; in two participants (one control subject and one hematological patient), the volume of whole blood collected was insufficient for analysis. Two participants returned invalid results and were excluded from further analyses. All 183 subjects could be tested with the Euroimmun test. Regarding the Roche Elecsys® assay, 151/179 subjects with readable results (84.3%) had detectable IFN-γ in plasma (median, 0.60 IU/ml; IQR 0.24–1.68) and the remaining 28 participants tested negative; regarding the Euroimmun test, 127/183 (69%) of participants were found to be reactive (median IFN-γ, 1.71 IU/ml; IQR 0.75–2.98), whereas 48 (26%) and 8 (4%) yielded borderline or negative results. As to the performance of the IGRAs across the study groups, the percentage of positive results returned by the Roche Elecsys® assay was 100%, 92%, 55%, and 100% for participants in Groups 1,2,3, and 4 respectively; whereas the percentages for the Euroimmun test were 96%, 22%, 76%, and 89%, respectively.

Of the 171 participants yielding interpretable results by both IGRAs (thus excluding those giving borderline results with the Euroimmun test and invalid results with the Roche Elecsys® assay), 150 yielded concordant results (positive, n = 124; negative, n = 26), and 21 were discordant. All but one discordance were Roche Elecsys® assay positive/Euroimmun test negative. These were more common among participants in Groups 2 (n = 10) and 3 (n = 8) than in Groups 1 and 4 (n = 1, in each group). The Median IFN-γ value in Roche Elecsys® positive/ Euroimmun test negative specimens was very low (0.077 IU/ml; IQR, 0.03–0.17). The Roche Elecsys® negative/Euroimmun test positive sample had an IFN-γ concentration of 260 mIU/ml (0.26 IU/ml). Consequently, overall, the PPA and NPA values across IGRAs were 86% and 55%, respectively, and Cohen’s kappa coefficient was 0.64 (95% CI 0.50–0.78). Of note, higher PPA, NPA, and Kappa values were observed in SARS-CoV-2-experienced individuals compared with SARS-CoV-2-naïve (Table 1). Importantly, PPA was lowest in patients from Group 2 (Table 1). ROC analysis indicated that establishing a lower cut-off for positive results in the Euroimmun test (45 mIU/ml; sensitivity, 90%, specificity, 89%) resulted in an improvement in PPA and Kappa values in all participant groups, including across both SARS-CoV-2-naïve and experienced individuals (Table 2).

**Table 1:** Qualitative performance comparison of the Roche Elecsys® IGRA SARS-CoV-2 assay and the Euroimmun SARS-CoV-2 test using the manufacturer’s respective cut-offs for positivity

Parameter	Overall	SARS-CoV-2 Infection status of participants^a^		Study group ^b^			
		Naïve	Experienced	Group 1	Group 2	Group 3	Group 4
PPA (%)	86%	67%	88%	98%	52%	80%	98%
NPA (%)	55%	36%	58%	NA	70%	25%	NA
Kappa coefficient [95% CI]	0.64 [0.50–0.78]	0.36 [-0.04–0.76]	0.67 [0.51–0.84]	NA	0.18 [-0.05–0.41]	0.32 [-0.08–0.72]	NA

NA, not applicable; NPA, negative percent agreement; PPA, positive percent agreement

^a^According to the absence (naïve) or presence (experienced) of detectable anti-SARS-CoV-2 Nucleocapsid total antibodies. For these analyses, a total of 25 SARS-CoV-2-naïve and 138 experienced participants were included

^b^Group 1 (n = 52), healthy controls; Group 2 (n = 44), hematological patients; Group 3 (n = 49), patients with chronic kidney disease or kidney transplantation; Group 4 (n = 26), elderly nursing home residents

**Table 2:** Qualitative performance comparison of the Roche Elecsys® IGRA SARS-CoV-2 assay and the Euroimmun SARS-CoV-2 test using a cut-off of 45 mIU/ml for the latter assay

Parameter	Overall	SARS-CoV-2 Infection status of participants^a^		Study group^b^			
		Naïve	Experienced	Group 1	Group 2	Group 3	Group 4
PPA (%)	88%	70%	91%	100%	58%	85%	100%
NPA (%)	58%	30%	63%	NA	68%	22%	NA
Kappa [95% CI]	0.68 [0.53–0.82]	0.29 [-0.15–0.74]	0.73 [0.58–0.88]	NA	0.55 [0.32–0.79]	0.29 [-0.20–0.78]	NA

NA, not applicable; NPA, negative percent agreement; PPA, positive percent agreement

^a^According to the absence (naïve) or presence (experienced) of detectable anti-SARS-CoV-2 Nucleocapsid total antibodies. For these analyses, a total of 26 SARS-CoV-2-naïve and 144 experienced participants were included

^b^Group 1 (n = 53), healthy controls; Group 2 (n = 49), hematological patients; Group 3 (n = 49), patients with chronic kidney disease or kidney transplantation; Group 4 (n = 28), elderly nursing home residents

### Quantitative performance comparison between the Roche Elecsys® IGRA SARS-CoV-2 assay and the Euroimmun SARS-CoV-2 IGRA test

**Fig. 1:**
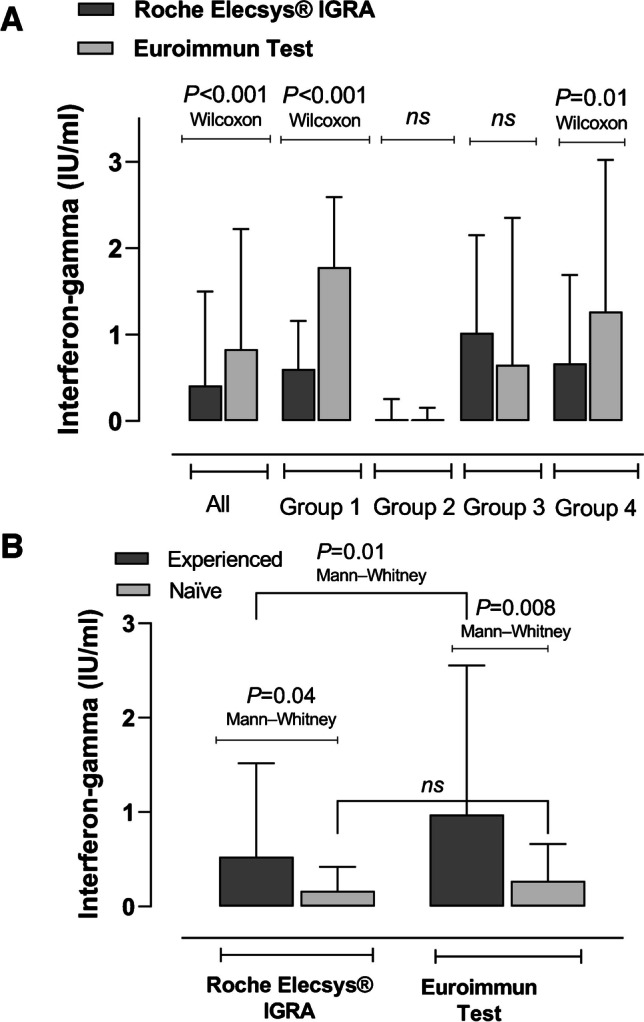
Quantitative results (IFN-γ concentrations) returned by the Roche Elecsys® IGRA SARS-CoV-2 immunoassay and the Euroimmun SARS-CoV-2 test according to (**A**) the participant population and (**B**) SARS-CoV-2 infection status. Group 1, healthy controls; Group 2, hematological patients; Group 3, patients with chronic kidney disease or kidney transplantation; Group 4, elderly nursing home residents. *P* values for comparisons, as determined by the Mann–Whitney U test or the Wilcoxon test, as indicated, are shown. Ns, not significant

**Fig. 2:**
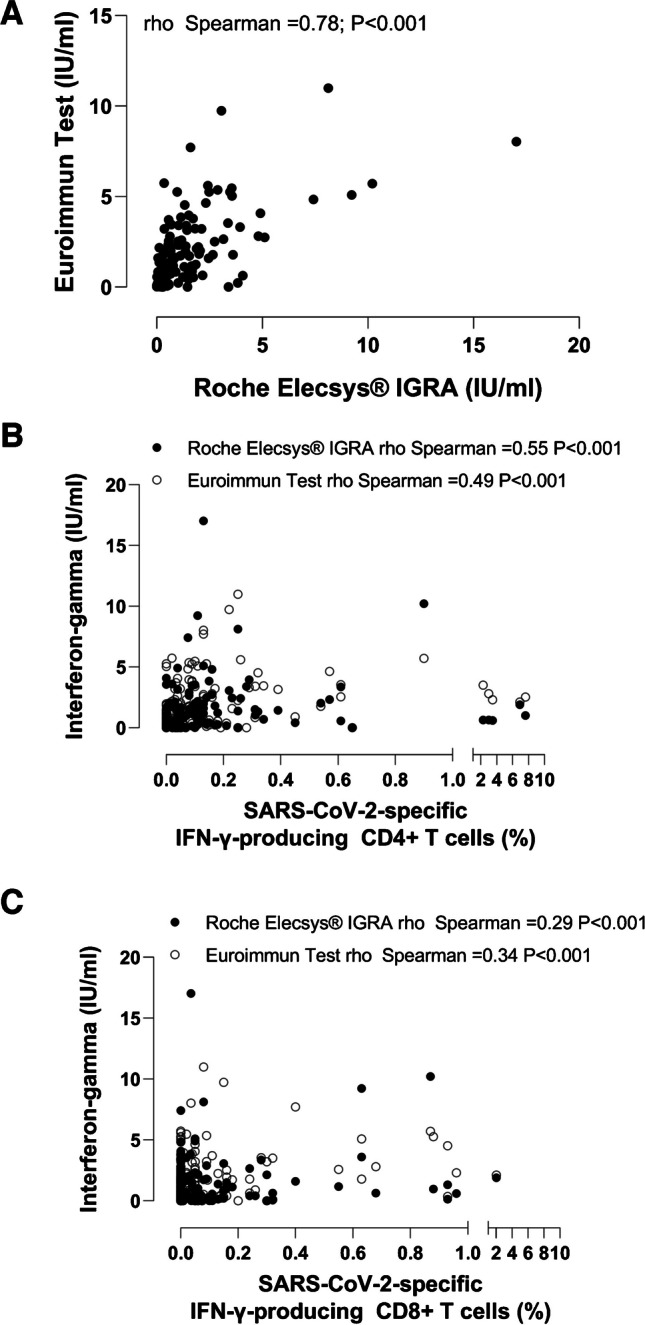
Correlation between quantitative results returned by the immunoassays compared. **A** Correlation between IFN-γ concentrations measured by the Elecsys® IGRA SARS-CoV-2 assay and the Euroimmun SARS-CoV-2 IGRA. **B** Correlation between IFN-γ concentrations measured by the Elecsys® IGRA SARS-CoV-2 assay and the Euroimmun SARS-CoV-2 IGRA, and frequencies (%) of IFN-γ-producing SARS-CoV-2-S-reactive CD4^+^ T cells. **C** Correlation between IFN-γ concentrations measured by the Elecsys® IGRA SARS-CoV-2 assay and the Euroimmun SARS-CoV-2 IGRA, and frequencies (%) of IFN-γ-producing SARS-CoV-2-S-reactive CD8^+^ T cells. Rho and *P* values (Spearman Rank correlation test) are shown

As shown in Fig. 1 (Panel A), IFN-γ concentrations quantified by the Euroimmun test were higher overall than those measured by the Roche Elecsys® assay (*P* < 0.001 by the Mann–Whitney U test). The difference was statistically significant for participants in Groups 1 (*P* < 0.001) and 4 (*P* = 0.01) but not for participants from Groups 2 and 3. SARS-CoV-2-experienced participants exhibited higher levels of IFN-γ irrespective of the IGRA used (Fig. 1, panel B). Overall, the correlation between IFN-γ concentrations measured by both IGRAs was strong (rho = 0.78; 95% CI 0.71–0.84; *P* < 0.001 by the Spearman rank test) (Fig. 2, Panel A). The degree of correlation was quite comparable across groups (Group 1, Rho = 0.70, 95% CI, 0.51–0.83, *P* < 0.001; Group 2, rho = 0.70, 95% CI 0.50–0.83, *P* < 0.001; Group 3, Rho = 0.68, 95% CI, 0.47–0.82, *P* < 0.001; and Group 4, Rho = 0.72, 95% CI, 0.45–0.88, *P* < 0.001). Interestingly, while the correlation was strong in SARS-CoV-2-experienced individuals (Rho = 0.76; 95% CI, 0.67–0.83; *P* < 0.001), it was moderate in SARS-CoV-2-naïve (Rho = 0.58; 95% CI, 0.22–0.80; *P* = 0.002).

### Qualitative and quantitative performance comparison between the flow cytometry immunoassay and IGRA tests

FC-ICS was performed in 179 participants, of which 135 (75%) tested positive, either displaying detectable CD4^+^ T cells (n = 58; 32%), CD8^+^ T cells (n = 18; 10%), or both (n = 59; 33%). In detail, 50/55 (90%), 26/50 (52%), 41/50 (82%), and 22/28 (79%) of participants from Groups 1, 2, 3, and 4, respectively, had detectable IFN-γ-producing T cells. Interpretable results from the three immunoassays were available from 177 individuals (Table 3). A total of 121 patients returned concordant results from all three immunoassays (positive, n = 104; negative, n = 17), whereas 56 patients had discordant results; the most common patterns of discrepant results across the assays were as follows: FC-ICS negative/Elecsys® IGRA and Euroimmun tests positive (n = 17), FC-ICS negative/Elecsys® IGRA positive/Euroimmun test negative or borderline (n = 8), FC-ICS positive/Elecsys® IGRA positive/Euroimmun test negative or borderline (n = 12), and FC-ICS positive/Elecsys® IGRA negative/Euroimmun test negative or borderline (n = 9). The PPA and NPA between FC-ICS and the Elecsys® IGRA were 78% and 34%, respectively (Kappa = 0.39), and 73% and 31% (Kappa = 0.42) between FC-ICS and the Euroimmun test.

**Table 3:** Qualitative performance comparison between the SARS-CoV-2 flow cytometry immunoassay and the Roche Elecsys® IGRA SARS-CoV-2 and Euroimmun SARS-CoV-2 test

Results of the IGRAs	SARS-CoV-2 flow cytometry immunoassay results		Total (%)
	Positive (n = 134)	Negative (n = 43)	
Euroimmun test Positive. no	105	17	122 (69%)
Roche Elecsys®-IGRA Negative. no	1	0	1 (1%)
Roche Elecsys®-IGRA Positive. no	104	17	121 (99%)
Euroimmun testNegative. no	22	25	47 (27%)
Roche Elecsys® IGRA Invalid. no	1	0	1 (2%)
Roche Elecsys® IGRA Negative. no	9	17	26 (55%)
Roche Elecsys® IGRA Positive. no	12	8	20 (43%)
Euroimmun test Borderline. no	7	1	8 (4.5%)
Roche Elecsys®-IGRA Negative. no	0	1	1 (13%)
Roche Elecsys®-IGRA Positive. no	7	0	7 (88%)

IGRA, interferon-γ release assay

As shown in Fig. 2 (panels B and C), the correlation between the frequencies of SARS-CoV-2-reactive IFN-γ T cells as quantified by FC-ICS and IFN-γ concentrations measured by IGRAs was moderate for CD4^+^ T cells: rho = 0.55 (95% CI, 0.43–0.64; *P* < 0.001 by the Spearman rank test) for the Elecsys® IGRA and rho = 0.49 (95% CI, 0.37–0.60; *P* < 0.001) for the Euroimmun test; and weak for CD8^+^ T cells: rho = 0.29 (95% CI, 0.14–0.42; *P* < 0.001) for the Elecsys® assay and rho = 0.34 (95% CI, 0.20–0.47; *P* < 0.001) for the Euroimmun test. Regardless of the patient group and the IGRA considered, Rho values were moderate at best for correlations with frequencies of IFN-γ-producing CD4^+^ T cells and weak, or very weak for IFN-γ-producing CD8^+^ T cells (Table 4).

**Table 4:** Correlation between frequencies of SARS-CoV-2-reactive CD4^+^ and CD8^+^ T cells as determined by flow cytometry for intracellular cytokine (IFN-γ) staining and IFN-γ levels measured by the Roche Elecsys® IGRA and Euroimmun test according to SARS-CoV-2 infection status and participant group

Participants	Roche Elecsys® IGRA		Euroimmun test	
	IFN-γ-producing CD4^+^ T cells Rho value (95% CI)/*P* value^a^/no. of participants	IFN-γ-producing CD8^+^ T cells Rho value (95% CI)/*P* value^a^/ no. of participants	IFN-γ-producing CD4^+^ T cells Rho value (95% CI)/*P* value^a^/ no. of participants	IFN-γ-producing CD8^+^ T cells Rho value (95% CI)/*P* value^a^/ no. of participants
All	0.55 (0.43–0.64)/ < 0.001/176	0.29 (0.14–0.42)/ < 0.001/176	0.49 (0.37–0.60)/ < 0.001/179	0.34 (0.20–0.47)/ < 0.001/179
SARS-CoV-2-Naïve^b^	0.46 (0.07–0.73)/0.02/ 26	-0.21 (-0.56–0.20)/0.30/ 26	0.14 (0.26–0.49)/0.49/ 27	-0.17 (0.52–0.23)/0.40/ 27
SARS-CoV-2-Experienced^b^	0.56 (0.43–0.67)/ < 0.001/142	0.34 (0.18–0.48)/ < 0.001/142	0.53 (0.39–0.65)/ < 0.001/142	0.35 (0.19–0.49)/ < 0.001/142
Group 1^c^	0.39 (0.12–0.61)/0.005/50	0.38 (0.10–0.60)/0.007/50	0.48 (0.23–0.67)/ < 0.001/51	0.36 (0.08–0.58)/0.01/51
Group 2^c^	0.57 (0.34–0.74)/ < 0.001/49	0.28 (-0.01–0.53)/0.053/47	0.60 (0.39–0.76)/ < 0.001/50	0.34 (0.05–0.57)/0.02/48
Group 3^c^	0.43 (0.16–0.64)/0.002/49	0.23 (-0.06–0.49)/0.11/49	0.28 (-0.003–0.53)/0.05/50	0.27 (-0.02–0.51)/0.06/50
Group 4^c^	0.59 (0.27–0.79)/0.001/28	0.08 (-0.31–0.45)/0.69/28	0.48 (0.12–0.73)/0.01/28	0.06 (-0.33–0.44)/0.75/28

IGRA, Interferon-γ release assay

^a^As determined by the Spearman Rank test; *P* values < 0.05 were deemed significant

^b^According to the absence (naïve) or presence (experienced) of detectable anti-SARS-CoV-2 Nucleocapsid total antibodies

^c^Group 1, healthy controls; Group 2, hematological patients; Group 3, patients with chronic kidney disease or kidney transplantation; Group 4, elderly nursing home residents

Finally, no significant differences for both SARS-CoV-2 IFN-γ-producing CD4^+^ and CD8^+^ T cells frequencies were observed between SARS-CoV-2-experienced and naïve participants: experienced—median 0.04% [IQR 0%-0.13%] vs. naïve—median 0.03% [0%-0.11%] for CD4^+^T cells (*P* = 0.97 by the Mann Whitney U test); median 0% [IQR 0%-0.07%] vs. median 0% [IQR 0%-0.02%], respectively, for CD8^+^ T cells (*P* = 0.18).

## Discussion

Here, we compared the performance of the Roche Elecsys® IGRA, the Euroimmun SARS-CoV-2 test, and an adapted protocol of the SARS-CoV-2 T Cell Analysis Kit from Miltenyi Biotec (FC-ICS) for the detection and measurement of SARS-CoV-2 T-cell responses. As a major strength, our study included several groups of vaccinated individuals differing in their net state of immunocompetence, which we hypothesized could have an impact on assay performance. The Roche Elecsys® assay offers a major operational advantage over other commercialized IGRAs by automating a crucial part of the testing workflow, the quantification of IFN-γ, which can be completed in around 20 min. To our knowledge, the performance of this assay has not been assessed. Major observations of our study are as follows. First, the qualitative agreement between both IGRAs was substantial (Kappa, 0.64), whereas that of FC-ICS (considering CD4^+^, CD8^+^ T-cell responses, or both) with either of the IGRAs was moderate at best. Overall, the Roche Elecsys® assay appeared to be more sensitive than the Euroimmun test, in that it returned more positive results; in this sense, notably, discordant Roche Elecsys® assay positive/Euroimmun test negative results were more frequently reported (51% vs. 22%) among hematological patients (Group 2). The sensitivity of the Euroimmun test could be slightly increased by lowering the cut-off for positivity (45 mIU/ml). In turn, the Roche Elecsys® assay was more sensitive than FC-ICS in all participant groups except Group 2 (positive results: 51% vs. 52%). The most plausible explanation for the increased sensitivity of the Roche Elecsys® assay compared with the Euroimmun test and FC-ICS is that the Roche Elecsys® assay can detect broader SARS-CoV-2 T-cell responses; it does not only include those targeting the S protein, like the Euroimmun test and FC-ICS, but also those directed against other structural and non-structural proteins. Additionally, that most participants were SARS-CoV-2-experienced also accounts for the greater sensitivity. Finally, both the Euroimmun test and FC-ICS detect SARS-CoV-2-S-reactive IFN-γ-producing T cells, however, the latter appeared slightly more sensitive overall (FC-ICS: 75% of positive results vs. Euroimmun test: 69%). Second, IFN-γ concentrations quantified by the Euroimmun test were generally higher than those measured by the Roche Elecsys® assay, although statistical significance was only observed for Groups 1 and 4. This may reflect differences in assay design and calibration and it is uncertain whether this may have any clinical relevance. Third, the correlation between IFN-γ concentrations quantified by IGRAs was strong and fairly similar across study groups. In contrast, the correlation between the frequencies of SARS-CoV-2-reactive IFN-γ T cells as enumerated by FC-ICS and IFN-γ concentrations measured by IGRAs was moderate for CD4^+^ T cells and weak for CD8^+^ T cells, regardless of the study group. Since we do not know the precise nature of the stimulating antigen in the IGRAs evaluated herein, no speculation can be made on this issue. Fourth, the Roche Elecsys® assay returned considerably fewer unreadable results than the Euroimmun test (2 vs. 28). In this sense, lowering the cut-off for positivity of the Euroimmun test to 45 mIU/ml may partially solve this issue. Fifth, SARS-CoV-2-experienced participants exhibited stronger T-cell responses compared with SARS-CoV-2-naïve subjects, as measured by IGRAs but not as determined by FC-ICS; the above observation further supports the assumption that individuals displaying hybrid immunity develop more robust adaptive immune responses than their vaccinated counterparts [15]. In this sense, the development of stronger S-targeted T-cell responses in SARS-CoV-2-experienced individuals may account for the higher qualitative agreement across IGRAs in these subjects compared with naïve individuals.

Prior studies comparing the performance of different commercially available IGRA, such as the Euroimmun test, the ELISpot-based assay T.SPOT-COVID (Oxford Immunotec, Abingdon, UK), or the QuantiFERON® SARS-CoV-2 IGRA (Qiagen, Hilden, Germany) and laboratory-developed flow-cytometry-based immunoassays for assessing SARS-CoV-2 T-cell immunity revealed striking differences in the rate of detection of detectable responses, the magnitude of the responses quantified, or both [11–14]. Importantly, these studies recruited seemingly healthy individuals (health workers) who had been fully vaccinated but did not include immunosuppressed or elderly subjects.

The current study has several limitations that must be acknowledged. First, the number of SARS-CoV-2-naïve participants was low. This may have undermined the robustness of analyses comparing the performance of immunoassays in this population group. Second, no data was available regarding either the precise time at which SARS-CoV-2 infection was acquired in SARS-CoV-2-experienced participants, whether reinfections occurred, or how SARS-CoV-2 infection evolved (clinical outcomes). Third, we assumed all assays to display 100% specificity, which may not be the case. Fourth, some participants could have been miscategorized as to their SARS-CoV-2-infection status. Fifth, although commercially available, the FC-ICS assay used lacks appropriate standardization.

In conclusion, here we have demonstrated that the Roche Elecsys® assay displays an overall increased sensitivity for the detection of SARS-CoV-2 T cells, compared with the Euroimmun test and the FC-ICS immunoassay used herein, in individuals displaying different degrees of immunocompetence. In this sense, the Roche Elecsys® assay may be particularly suited to revealing low-magnitude T-cell responses that may be present in immunosuppressed individuals, such as hematological patients. The study also stresses the fact that the correlation between quantitative values provided by different SARS-CoV-2 T-cell assays may vary significantly; this observation should be taken into consideration in studies aimed at defining thresholds for protection against SARS-CoV-2 infection and COVID-19.

## Data Availability

The datasets generated and/or analyzed during the current study are available from the corresponding author on reasonable request.
